# Commentary: A new era in the management of spinal metastasis

**DOI:** 10.3389/fonc.2024.1494078

**Published:** 2024-12-19

**Authors:** Denis Aiudi, Mauro Dobran

**Affiliations:** Neurosurgical Department, Marche Polytechnic University, Ancona, Italy

**Keywords:** spinal, metastases, vertebral, tumor, neurosurgery

## Introduction

In spinal tumor metastases the gold standard treatment is surgery, followed by chemo and radioterpy (1).

Surgery remains the fulcrum of the therapy, especially in cases with spine instability and spinal cord involvement. In literature, the debate regarding the feasibility of the decompressive treatment and its optimal timing for surgery is still open and it is very difficult to determine the optimal surgical timing in patients with vertebral metastases starting with a neurological deficit (2). We strongly believe that surgery timing is an important prognostic factor for the clinical outcome in patients treated for spinal metastases with acute neurological deficit. As reported in our study regarding the cervical spinal cord injury (3) the surgical timing is relevant also for the spinal compression in vertebral metastases.

In our previous study we considered 81 patients with traumatic cervical spinal cord injury operated before and after 12 hours. Forty seven of 81 (58%) patients exhibited improved neurological function and 72.% of them was treated <12 hours after the injury. This ultra-early surgical timing in this type of patients was also associated with significantly greater neurological improvement.

## Discussion

In our experience, spinal metastases arising with acute neurological deficit can benefit from early surgical treatment within 12 hours of onset, with a satisfactory neurological recovery, as for traumatic pathology. A statistical analysis of the data was performed, in order to demonstrate any significant difference in the neurological outcome of the patients operated on with different timing. For this purpose, the analysis of variance system ANOVA was used, in order to compare differences between certain groups. A result with p-value < 0.05 was considered significant. Through statistical analysis, a statistically significant difference (p < 0.05) was found, in terms of neurological outcome, between those who were operated on within 12 hours of the onset of symptoms and patients operated on later. In fact, all subjects operated on within 12 hours had a clinical improvement, assessed by Frankel grade.

These results are in agreement with the current literature that confirm the usefulness of an early approach in these patients. In the series of Meyer et al. (4) the best clinical results were obtained in less than 16 hours from the starting of neurological deficit. B. K. Kwon et. al (5) in their paper hypotized that, knowing the pathophysiology of the acute spinal cord damage, an early management of the tumor interrupt the cascade of events responsible of the neurological secondary injury with better residual spinal cord function. Our study confirm these results, surgery within 12 hours from the onset of neurological deficit in patients with vertebral metastases offers a chance to preserve the neurological residual functions. This data was previously confirmed also in spinal cord injury (3).
